# Possibility of Inflammation in Cold-Blooded Animals

**Published:** 1845-06

**Authors:** 


*Possibility of Inflammation in Cold-Blooded Animals.*—M. Sereboullet, of Strasbourg, informed the Academy, that while examining a cayman, which had died in a travelling menagerie, he found that death had been caused by extremely intense peritonitis. The peritoneum was highly vascular, and coated, throughout its entire extent, with pus, which lay in false membranes that could be peeled off in flakes. The intestines were of a wine-red colour, and agglutinated together by false membranes.

On separating the intestines from each other, a piece of cork was found in the peritoneal cavity; the perforation of the intestine through which it had escaped was almost linear, and was situated in the angle of the fourth fold of the intestine. In this instance the characters of true inflammation were present,—intense redness, exudation of plastic lymph, formation of false membranes, adhesions, secretion of pus. It is thus shown, beyond all doubt, that inflammation can occur in cold-blooded animals.—*Gazette Medicale de Paris.*

